# Dietetics1Read before the American Veterinary Medical Association, New York, September, 1899.

**Published:** 1899-10

**Authors:** W. H. Dalrymple

**Affiliations:** Baton Rouge, La.


(To be continued.)

---

### DIETETICS.[1]

By W. H. Dalrymple, M.R.C.V.S.,

BATON ROUGE, LA.

The thought has on several occasions occurred to me that the subject of veterinary dietetics would be both an interesting and appropriate one to present before our national body. Why, I cannot quite explain; but I seem to have been led to the conviction that as a profession, we, in our strenuous efforts along the lines of pathological and therapeutical research, were somewhat apt to fall

[1] Read before the American Veterinary Medical Association, New York, September, 1899.

into the error of losing sight of, or overlooking, some of the great physiological principles which form the basis of a perfect bodily condition and the nutritive requirements for its maintenance. With our enormous wealth in livestock in this country, and an increasing tendency to improvement in the various breeds of animals, there is no branch of our professional work that can be of greater importance than that of dietetics. Feeding! That seems a very common-place expression; but what a wonderful expanse of territory it covers. Compared with the human dietitian, the veterinarian has a much more extended field. In the first place, the former, having only one set of digestive organs to deal with, is rarely sought after for advice, except in the case of a dyspeptic, or in convalescence after sickness; but, apart from the stereotyped forms of easily digestible foods suggested by the veterinarian during recovery of his patient from some debilitating illness, he may be asked —and he ought to be able to furnish an intelligent answer—as to the food requirements of the different domestic animals for maintenance, for growth and development, for work of all kinds, for milk, for wool, etc. And in his case it is much more difficult, having to deal with at least three different arrangements of digestive apparatus. How, then, is he to be able to get an intelligent grasp of this subject and place himself in the position where he can be not only of value to his client as a conserver of the health of the livestock, but of his wealth as well? It seems to me that a more extended knowledge of this kind would enhance the value of the services of the veterinarian many fold. True, it may be claimed that the veterinarian is rarely, if ever, asked for information regarding the nutritive value of the various food-stuffs, or how to properly balance a ration for a specific purpose; but if we all possessed the necessary and accurate knowledge on this subject, the stock-owning public would no doubt make more use of us, and to our mutual benefit.

In order to have a somewhat clear understanding of our subject, it might be well for us first of all to view the animal body as a machine, and in doing so, we will consider it under two heads, viz., income and expenditure. In the first place, we have income of potential energy. This consists of potential energy in food, minus the potential energy left in the waste products, such as urea, expended by the kidneys; carbonic acid by the lungs, and nitrogen in the excreta. Then we have potential energy by oxidation in the tissues, said to be most in muscle, next in glands, as the liver, etc. —potential energy being liberated as kinetic energy. The kinetic energy which is expended is said to consist of heat, five-sixths, and mechanical work, one-sixth. The distribution of animal heat is effected by the circulating blood, and the temperature of warm-blooded animals is regulated mainly by increasing or diminishing loss of heat by the skin; the vasomotor nerves dilating the arteries, thus increasing loss of heat, or constricting them, diminishing loss. Heat is gained, first, by oxidation in the tissues; second, by friction; and third, by ingestion of hot food and liquids. Heat is lost by, first, the skin—by radiation, conduction, and evaporation; second, by the lungs—by evaporation and by warming expired air; and, third, by ejections of warmed feces and urine.

Now, that this machine should work smoothly and without a hitch, it is essentially necessary that the food be of sufficient quantity and of the best quality, also that the sanitary arrangements and surroundings generally be as perfect as possible. To take a familiar illustration: The steam-engine requires a great deal more than the mere piling on of fuel; it must also be kept thoroughly clean. Every piston and crank must work with perfect smoothness, and every valve clear, so as to allow of the escape of superfluous steam. The better the quality of the fuel and oil, the more harmoniously will the parts work. If too much fuel is put on, there is the risk of damage to the machinery; if too little, the machine stops. So it is with the animal machine. It requires careful and intelligent attention; neither too much nor too little food, but the requirements should be of sufficient quantity and of a desirable quality. These are, of course, physiological facts and known to you all.

In considering the income and expenditure of food we have, of course, food and water taken in by the mouth; oxygen by the lungs and skin, minus the feces. On account of expenditure, which includes excreta and waste-products, we have, first, nitrogen expended by the kidneys, as urea, etc.; second, carbon, by the lungs and skin, as carbon dioxide; third, minerals by the kidneys; and, fourth, water, by the lungs, skin, and kidneys.

The foods required by the animal economy are made up into four groups or classes, similar, it may be said, to those entering into the composition of the tissues of the body, but with the addition of a class known as carbohydrates, found in the food of the herbivora.

The first group is known as nitrogenous, proteid, protein, sometimes albuminoid, although this is, correctly speaking, one group of the protein compounds, the other being the amides. Examples are albumin, glutin, legumin, found in flesh, grain, peas, etc. As to their destiny, they are built up into tissue-proteids for the building up and repair of the working machinery of the body, and to supply the necessary protein for the production of milk, etc., or they are split up, one part being oxidized and excreted as urea, etc., and a part changed into fat and stored up. Liebig held that protein substances were wholly built up into proteid tissue, such as muscle, and then oxidized to provide mechanical work; but if in excess of the amount required to build up and repair the waste of them the protein may be converted into fat and deposited as such, or used to produce heat and energy. In other words, provide both mechanical work and heat.

The second class is the fats, hydrocarbons, or as they are now more familiarly known, ether extract. Examples of these are palmatin, olein, and stearin. These contain very little oxygen, therefore their carbon and hydrogen are largely available for oxidation. These substances are first oxidized and excreted as water and carbon-dioxide; second, changed into other forms of fat and stored up. Liebeg held that part of the fat was oxidized to provide heat only; but it is considered by other authorities that fat is oxidized to provide both mechanical work and heat, and that it is doubtful if any is stored. For we are told that while exclusive protein feeding induces only protein consumption in the body, and does not contribute to the formation of flesh, an exclusive fat diet has no influence on the deposition of fat in the body, the same amount being deposited whether much or little fat is fed. A dog experimented with by Pettenkofer and Voit lost 96 grammes of fat daily during hunger. When 100 grammes of fat were fed daily an average of 97 grammes of fat was oxidized, showing that the loss of fat in the body was barely covered by the feeding of fat.

The third class are the carbohydrates, which include two groups: Nitrogen free extract, such as starch, sugar, gum, etc., and fibre or the woody part of plants. The first are easy of digestion; the second much less so, although that digested fulfils a like function. The carbohydrates are converted into some form of sugar, which enters the blood by absorption from the intestinal canal. In the liver, the sugar is converted into glycogen. Their destiny is oxidation into water and carbon-dioxide, and as in the case of protein and fats, their oxidation provides both mechanical work and heat. The carbohydrates constitute the largest part of vegetable foods. There is no starch in the animal body, consequently they are not stored up as such.

The fourth class comprises minerals and water. The mineral matter is found in what is known to the chemist as ash, which is the residue left after the organic matter has been driven off from a food-stuff by heat. This is composed largely of calcium, magnesium, potassium, sodium, and iron salts, chlorine, and sulphuric, carbonic, and phosphoric acids. The ash of the food is the source from which the mineral matter of the animal body is obtained, and as such is very important.

No matter how dry any food-stuff may appear, it nevertheless contains a considerable amount of water. Water in grains and dried fodders ranges from 8 to 15 per cent. of the whole; in green forage and silage is about 80 per cent., while in some roots it runs as high as 90 per cent. Computations are not based, however, on the contained water in food-stuffs, but upon the water-free or dry matter content.

As to composition, then, foods are divided into two great classes, viz., carbonaceous or non-nitrogenous, including all those which do not contain nitrogen. These, again, are divided into two classes: (*a*) Carbohydrates, or those in which carbon predominates, and (*b*) hydrocarbons, or those in which hydrogen predominates, and nitrogenous. As to nutrition, they are also divided into two great classes, viz.: Calorifacient, or heat-making, and histogenetic, or tissue-building. In view of the fact that the materials of which the animal body is composed are being constantly broken down and consumed, to keep the animal alive and in a healthy condition this waste of body material must be made good by a constant supply of new material, which is one of the chief functions of food. But in addition to this, food, as we have already noted, serves other important functions. It serves to maintain the heat of the body, to furnish the energy which is necessary to enable the animal to move and to perform work, to promote growth by producing flesh and bone, to fatten, or produce milk, and so on.

We are all aware that the ultimate object of the greater portion of the food supplied to the animal is the production of work of some kind. All manifestations of life, shown in a thousand ways by the animal, are, as Henry says, in some manner derived from the food, but in what way the food is converted into energy will always exceed man's power to definitely determine. It is a fact, however, that the question of "how to feed" our animals so as to get the best possible results at the minimum of cost is one of the economic problems of the day, and must be based upon a scientific foundation. And although it may be surrounded by metaphysical intricacies which it may never be within our power to penetrate, still science has revealed to us many facts regarding animal nutrition that are extremely helpful, and which can be successfully utilized along practical lines.

One of the first essentials of a food should be purity. This is a point which should be very strongly impressed upon the minds of clients. There is a too prevalent idea with many stockowners that there is economy in cheapness; that anything is good enough for a hog, and that the superior digestive powers of the cow will render sterile and nutritious food-stuffs that are in reality only fit for the refuse pile. This suggests the necessity for education. I am not so familiar with the conditions existing in other parts of the country, but we in our section suffer keenly for lack of correct knowledge on the subject of foods and feeding. We purchase food-material from the North, East, and West that would be hard to dispose of on the markets in those sections. I do not believe it is with the object of economizing, for we pay top prices. It is because we are as yet not educated up to the point of being able to properly discriminate; but we are improving. It is a very important matter, then, that food should be pure and from healthy and unadulterated sources. It is unnecessary to go into the various agencies which render food-stuffs impure; I shall mention one, however, which is occasionally found, and often produces very fatal results. I refer to the spores of anthrax. I have on more than one occasion experienced outbreaks of the disease resulting from the food being contaminated with spores of the bacillus anthracis. For this reason I am always suspicious of manufactory-prepared or mixed feeds, as it is impossible to know where the grains have been raised from which they are made, and if from a charbonous district where sanitary measures have not been enforced for the proper disposal of carcasses, contamination of the food-plant and of its products is liable to take place.

The next essential is digestibility. This may be said to be the leading quality of foods. It is possible for us to take two foods which contain a similar amount of chemical elements, and which theoretically appear to be of the same value, but there may be a great deal of difference in their digestibility, and, consequently, in their worth as nutrients. The digestibility of a food is determined by the chemist by first analyzing to find the percentage of each of the nutrients it contains. Some animal is then fed with weighed quantities of the food, and the solid excrement voided during the trial is saved, weighed, and samples of it are analyzed. Having a knowledge of the amount of each nutrient fed, and knowing the quantity that reappears in the solid voidings, the difference is considered to be the digested portion, since it must have been retained within the body. There is no necessity nowadays for anyone interested in the subject of foods and feeding to be without the required information relative to their percentage composition and digestibility. Our numerous experiment stations have worked out for us just these very problems, and we have those valuable analyses compiled and issued by the United States Department of Agriculture, which are within the reach of everyone.

As all foods are not equally well balanced as to their nutritive relations, and as the large and varied capacities for work in the different parts of the digestive tract require foods of different nature to bring into activity all the digestive forces, it is essential that the proximate principles should be in the proportions commensurate with these requirements.

This brings us to a consideration of the "nutritive ratio" of a food, by which is meant the ratio that exists between the amount of the digestible protein in a given food-stuff and the amount of the digestible carbohydrates and ether extract it contains; or, in other words, the proportion in which the nitrogenous elements exist in relation to the fats and carbohydrates, minus the cellulose. This is ascertained by first of all multiplying the amount of digestible ether extract, or fat, by 2.4 or 2.25—the factor generally used in this country—its heat value compared with the carbohydrates. The product is then added to the total quantity of digestible carbohydrates, and the sum is divided by the digestible protein. If the ratio between the nitrogenous and the non-nitrogenous elements is less than 1 to 5, it is said to be narrow or high; if greater than 1 to 6, it is looked upon as wide or low. For instance, if we take alfalfa or lucerne, we find that it has a nutritive ratio of a little over 1 to 3. This is narrow, and is rich in albuminoids compared to its non-nitrogenous constituents, which would suggest its value as a food for growth and development, or to balance a mixed ratio the other ingredients of which contain an excess of carbohydrates and fat. Oat straw, on the other hand, has a nutritive ratio of 1 to 33, which is a good illustration of a wide ratio, and would indicate the necessity of feeding a more concentrated food along with it in order to more evenly balance the proximate principles of the ration.

While, however, the work of the physiologist and chemist has been proceeding in the line of analysis and digestion experiments, feeding-trials have also been in operation to determine the requisite amount of the different nutritive elements necessary for the proper nourishment of the various classes of the domestic animals of the farm. It would occupy too much time and space to go into the feeding standards for the conditions and wants of the different farm animals, but if we consult those of Wolff, modified by Lehmann, for horses weighing 1000 pounds, doing light work, we find that they require per day 20 pounds of dry matter, 1.5 pounds of protein, 9.5 pounds of carbohydrates, 0.4 pound of ether extract, giving a nutritive ratio of 1 to 7; while horses of the same weight and doing heavy work require 26 pounds of dry matter, 2.5 pounds of protein, 13.3 pounds of carbohydrates, and 0.8 pound of fat, this ration having a nutritive ratio of 1 to 6. It must be understood, of course, that a feeding standard varies with the kind of animal and the purpose for which it is kept.

Although the standards of Wolff are generally recognized as authoritative the world over, we occasionally come across some rather impressive anomalies. In looking over a very interesting article in an Australian journal, on the "Economic Feeding of Working Horses," read before the Congress of the Australian Association for the Advancement of Science by T. U. Walton, B.Sc., F.C.S., etc., chemist, I believe, to a large colonial sugar refining company in Fiji, I was very much struck with his result, more especially in the width of the nutritive from which he appeared to obtain the most satisfaction, both as regarded the health and working condition of the animals, as well as economy in the material fed. He begins by stating that the feeding trials, which were conducted on a very large scale, proved that, at least under certain conditions, a high diet is not essential to the performance of hard work or the maintenance of good condition, provided sufficient nutritive food be given. In Fiji the Colonial Sugar Refining Company has about 1000 head of farm horses, which, until a few years ago, were fed chiefly on oats and corn, with some green sugar-cane tops in addition. But this did not prove satisfactory in the trying tropical climate; sickness was frequent, and the death-rate high, while the charge for fodder was very heavy. As large quantities of waste molasses were available, it was thought well to investigate whether the sugar in this material might not be advantageously used as a substitute for some of the starch in the ordinary food. When these trials were commenced care was taken to begin with only small quantities of the new fodder, lest the high proportion of salts should prove too laxative. With the growing appetite for it the proportion of molasses was gradually increased till as much as thirty pounds per day were given regularly to many of the animals. This large proportion was after a time, however, reduced to fifteen pounds; not by reason of any ill effects beyond a tendency to fatten, but because it was considered too risky an experiment with so much valuable stock. Contrary to expectation, the molasses diet produced constipation, instead of being laxative, and a few pounds of bran had to be given daily to keep the bowels in order. The ration finally adopted was 15 pounds of molasses, 3 pounds of bran, and 4 pounds of corn per day, with as much green cane tops as the animal could eat, the molasses being mixed with the bran and chopped cane tops. This ration had been fed daily to over 400 head of horses (at the time the paper was read) for nearly two years, with the following results, which we quote from the article: There has been no undue fattening nor injury to the wind, and no tendency to excessive perspiration or softness. In the early stages of the trials a dozen of the animals were weighed once a month, the average weight at the start being 1273 pounds. After the first month there was an average loss of weight of 15 pounds per head; after the second month, 4.5 pounds of the loss had been recovered, and after the third month there was a further gain of 16.5 pounds, making a gain over the whole period of 6 pounds per head. Sickness, which formerly was frequent, is now uncommon, and the horses are capable of performing harder and more continuous work. The improvement in this respect is so great that while the area of cultivation has been largely increased, it has not been necessary to make any addition to the working stock. The conclusions which Mr. Walton thinks can be fairly drawn from the trials made are:

1. That for working horses the sugar in cane-molasses is a satisfactory substitute for starchy food, being readily digested and transformed into work.

2. That fifteen pounds of molasses can be given to a 1270-pound working horse, with advantage to the health of the animal and to the efficiency of its work.

3. That it produces no undue fattening, softness, nor injury to the wind.

4. That the high proportion of salts in it has no injurious effect.

5. That the albuminoid ratio as low (wide) as 1 to 11.8 has proved highly suitable for heavy continuous work when a sufficient quantity of digestible matter is given.

The following is the composition of the daily ration per 1000 pounds, live weight: 1.13 pounds digestible albuminoids; 13.31

pounds digestible carbohydrates (including 0.24 pounds of fat). Albuminoid ratio, 1 to 11.8.

From the above, as Mr. Walton states, it is thus seen that the full proportion of carbohydrates considered necessary by Wolff for a hard-working horse has been experimentally arrived at in these trials, but that only half the orthodox proportion of albuminoids has been found necessary, and only half the fat. Probably, he adds, the warmth of the tropical climate renders the small proportion of fat sufficient, but the satisfactory results obtained with the reduced proportion of albuminoids prove that the current theory on the matter is erroneous.

